# The Brazos Valley Medical Association

**Published:** 1897-12

**Authors:** W. B. Briggs

**Affiliations:** Sec. Brazos Valley Medical Association; Easterly, Texas


### THE BRAZOS VALLEY MEDICAL ASSOCIATION.

REPORT OF PROCEEDINGS: BY THE SECRETARY.

EASTERLY, TEXAS, Nov. 20, 1897.

The Brazos Valley Medical Association convened at the Columbia opera house in Navasota, at 10 a. m. on Tuesday, November 9. The introductory session was opened by music, selected by Prof. Brill's Ideal Mandolin and Guitar Club, which was composed exclusively of ladies. Invocation of divine blessings, by Rev. J. T. Sloan. Then a sweet solo, by Miss Lillian Shaw. Address of welcome, by Dr. D. L. Peeples, vice-president; address of welcome by Geo. D. Neal, in behalf of the mayor. Response by Dr. G. M. Abney, president. Many ladies were present at the exercises.

The meeting was called to order for business at 2:30 p. m., with President Abney in the chair. The following was the program on the occasion:

1. Placenta Prævia, by Dr. J. L. Hollman, of Franklin.
2. The use of cocaine as an anæsthetic, by Dr. H. W. Cummings, of Hearne.
3. Gun shot wounds, by Dr. E. N. Shaw, of Cameron.
4. Uterine displacements, by Dr. E. A. Harris, of Navasota.
5. Typhoid fever, by Dr. E. A. Thompson, of Navasota.
6. Tuberculosis of joints and bones, by Dr. D. L. Peeples, of Navasota. *
7. Total excision of larynx for intrinsic malarial disease, by Dr. J. A. Bodine, of San Antonio.

These papers were read and thoroughly discussed.

Twenty new members were added to the association.

By request, Dr. Bodine, of San Antonio, performed two operations for Intestinal Anastimosis. These were eagerly

* Published herewith.

watched by the members and all were delighted with the skill and ease with which these operations were performed.

Calvert was selected as the next place of meeting and the time, the second Tuesday and Wednesday in May. A grand banquet was tendered the physicians at the Knights of Pythias hall, which the doctors and their guests enjoyed to the fullest extent.

The following resolutions were unanimously adopted:

"Whereas, the extraordinary session of the Twenty-fifth Legislature passed a law imposing an annual occupation tax on every local practicing physician in this state;

Whereas, it is a thrust at a profession whose very foundation is charity. It is a weak confession on the part of our law makers that it must strike right and left to raise revenue by whatever means.

It is a tax wholly unjust because it seeks to place an embargo on the only profession known to "sweet charity."

Through the resources of its followers it has furnished more relief to suffering humanity than all other professions combined.

From the life of Sims, Gross, Jenner, Mott and McDowell there emits a halo of light like that which surrounds the brow of a saint.

When plagues, contagious and infectious diseases are threating the people the medical profession stands as a wall of protection for the public's good. The medical profession has erected for itself, on many a bloody battlefield, monuments that will stand while granite crumbles, and no man can shatter the urn on which are inscribed the memories of its illustrious writers.

Whereas, the profession of medicine is and should be considered the most sacred and noble calling, save the ministry; and whereas, the medical profession is looked to by the state, county and municipal governments, as well as by the individual, to guard their public health and give their services to all classes alike, disregarding pecuniary reward; And whereas, the better part of the profession of Texas have been, and are now, under unfavorable condition and against the prejudices of the ignorant, doing all in their power to elevate the standard of pure medicine in Texas; And whereas, the recent legislature has treated their efforts to raise the standard of medicine with indifference; And instead of placing the medical standard upon an educational plane, with other states, they have given the profession an insult by placing it among the ordinary trades, by requiring an occupation tax of insignificant value; therefore, be it resolved

1. That we condemn the last acts of the legislature, regarding the medical profession, in refusing to raise the qualifications of practicing physicians, and in still lowering the profession in the eyes of the world by making the noblest of all callings an ordinary trade.

2. Be it further resolved, That we pledge ourselves to actively oppose any candidate for the state assembly or legislature who will not pledge himself to support some good bill for the raising of the educational standard of medicine and repeal the act making an occupation tax upon physicians regularly qualified and practicing medicine.

3. Be it further resolved, That we condemn the action of the last speaker of the house, L. T. Dashiel, for blocking legislation, thereby defeating the passage of the State Association bill, which was intended for the good of the profession in Texas.

4. Be it further resolved, That we earnestly request every medical association and individual doctor in Texas to take some active part in securing such members to the next legislature who will appreciate our efforts to have a better standard of medicine in Texas and who will pledge their support to such a bill.

5. Be it further resolved, That we recommend the appointment of delegates from every association in the state to represent them in the next State Association and there promulgate such a bill as will in their wisdom subserve the interest of the profession, and that the state association send committees to represent our interests before the next legislature.

6. Be it further resolved, That a copy of these resolutions be furnished to the state papers and the TEXAS MEDICAL JOURNAL."

H. W. CUMMINGS, M. D.
J. F. EAVES, M. D.
E. A. HARRIS, M. D.
Committee.

W. B. BRIGGS, M. D.,
Sec. Brazos Valley Medical Association.

